# Mechanical and Thermal Characterization of Natural Intralaminar Hybrid Composites Based on Sisal

**DOI:** 10.3390/polym12040866

**Published:** 2020-04-09

**Authors:** Alexandre L. Pereira, Mariana D. Banea, Jorge S.S. Neto, Daniel K.K. Cavalcanti

**Affiliations:** Federal Center of Technological Education in Rio de Janeiro, CEFET/RJ, Rio de Janeiro 20271-110, Brazil; alexluizp@gmail.com (A.L.P.); danielkkc@gmail.com (D.K.K.C.)

**Keywords:** hybrid composite, intralaminar, mechanical characterization, thermal analysis, sisal, ramie, curauá

## Abstract

The main objective of this work was to investigate the effect of hybridization on the mechanical and thermal properties of intralaminar natural fiber-reinforced hybrid composites based on sisal. Ramie, sisal and curauá fibers were selected as natural fiber reinforcements for the epoxy matrix based composites, which were produced by the hand lay-up technique. Tensile, flexural and impact tests were carried out according to American society for testing and materials (ASTM) standards to characterize the hybrid composites, while differential scanning calorimetry (DSC) and thermogravimetric analysis (TGA) were used to evaluate the thermal properties. It was found that the mechanical properties are improved by hybridization of sisal based composites. The thermal analysis showed that the hybridization did not significantly affect the thermal stability of the composites. A scanning electron microscopy (SEM) was used to examine the fracture surface of the tested specimens. The SEM images showed a brittle fracture of the matrix and fiber breakage near the matrix.

## 1. Introduction

In the recent years, natural fiber-reinforced composites are seen as realistic alternatives to replace the synthetic- (i.e., glass) reinforced composites in many applications. The main reasons are the lower weight and relatively lower cost of natural fibers. However, natural fiber-reinforced composites vary greatly in their mechanical properties. The mechanical properties of composites (e.g., tensile, flexural and impact) are highly dependent on different factors such as fiber and matrix type, interfacial adhesion between fiber and matrix, fiber dispersion and orientation, processing, among others. By increasing their mechanical performance the capabilities and applications of natural fiber-reinforced composites can be greatly extended. One method to increase their performance is the hybridization. In this context, natural fiber-reinforced hybrid composites, which contain one or more types of fiber are gaining growing research interest [1,2,3,4,5,6]. Both natural and synthetic fibers can be used to produce hybrid composites [7]. For instance, the hybridization of natural/natural fibers in the composite can help due to the differences in cellulose content in the natural fibers, which has a significant influence on the mechanical properties of the composites. In addition, each fiber has its own distinctive characteristics necessary for a specific application. 

The fibers used in this work were Sisal (*Agave sisalana*), Ramie (*Boehmeria nivea*) and Curauá (*Ananas erectifolius*), as these vegetable fibers are widely available in Brazil and are ecologically sustainable and viable. The applications of natural fiber-reinforced composites have increased intensely in the last years. Some examples of applications of natural fiber-reinforced composites in industry are construction and furniture industry, music instruments, protective materials (shin guards and helmets) in the sports industry and secondary structures (such as door panels, dash boards, headliners and seat backs,) in the automotive industry [8,9,10,11,12,13].

Several researchers investigated the effects of hybridization on the mechanical and thermal properties of hybrid composites reinforced with different vegetable fibers (i.e., jute, curauá, sisal, ramie, coconut, sugar palm, etc.) or natural fibers combined with glass fibers [14,15,16,17,18,19,20,21,22,23,24,25]. For example, Almeida et al. [14] showed that hybrid composites in which 30 vol.% of glass fiber was replaced by curauá fiber was able to achieve similar results in terms of hardness, impact strength and dynamic mechanical properties. Ferreira et al. [15] investigated the mechanical properties of hybrid sisal/glass composites with inclusions of silica microparticles. The results showed an increase of tensile and flexural strength (by approximately 80%) of hybrid sisal/glass composite when compared to non-hybrid sisal composite. The incorporation of silica (5%) improves the mechanical performance of composites containing a larger volume of sisal fibers. Cavalcanti et al. [17] investigated the effect of hybridization on the mechanical properties of intralaminar hybrid epoxy composites based on jute. It was found that the tensile strength of jute+curauá and jute+sisal composites increased by approximately 77% and 68%, respectively, compared to non-hybrid jute composites. Giridharan [26] evaluated hybrid ramie/glass fiber (20%/80% and 30%/70%), epoxy composites by tensile, flexural and impact tests and found that the hybrid composites (30%/70%) exhibited better mechanical properties than non-hybrid composites. Chen et al. [27] examined the mechanical and water absorption behaviors of corn stalk/sisal fiber hybrid composites. It was showed that the hybridization with sisal fiber significantly enhanced the mechanical properties (flexural strength increased by 20% and tensile strength by 49.5%) but had little effect on water absorption. Boopalan et al. [18] studied the effect of hybridization on mechanical and thermal properties of jute and banana fiber-reinforced hybrid composites with different fiber percentages (100%/0%, 75%/25%, 50%/50%, 25%/75% and 0%/100%—jute and banana), by performing tensile, flexural, impact and thermal tests. It was found that addition of banana fiber in jute/epoxy composites of up to 50% by weight results in increasing the mechanical and thermal properties. Braga and Magalhães [23] studied the effect of hybridization on jute and glass fibers-reinforced composites and showed that hybridization had a positive effect on the mechanical properties (density, impact energy, tensile and flexural strength). Zin et al. [16] studied the thermal and mechanical properties of the hybrid composite of banana, pineapple and glass fiber reinforced epoxy matrix with different fiber volume fraction (30%, 40% and 50% in weight). Specimens of 40 wt % showed better flexural strength and offered optimum onset temperature degradation by TGA analysis.

Gupta and Srivastava [28,29] studied the dynamic mechanical and thermal properties of hybrid jute/sisal fiber reinforced epoxy composites and found a positive effect of hybridization in terms of dynamic mechanical and thermal properties. Storage modulus, loss modulus and *T*_g_ were found to be higher for hybrid composite having a higher percentage of jute fibers. Jarukumjorn and Suppakarn [30] investigated the thermal properties of glass/sisal fiber-reinforced polypropylene (PP) composite with different weight ratios of fibers and found that the decomposition temperature increased by increasing the glass fibers content in the composites.

Although, the effect of hybridization on the mechanical and thermal properties of natural fiber-reinforced composites was investigated by several researchers, most of the works were concentrated on interlaminar hybrid composites. However, the main objective of this work was to investigate the effect of hybridization on the mechanical and thermal properties of intralaminar natural fiber-reinforced hybrid composites based on sisal (i.e., Sisal+Ramie (S+R), Sisal+Curauá (S+C) and Sisal+Glass fiber (S+G) composites). Tensile, flexural and impact tests were performed for the mechanical characterization, while for the thermal analysis TGA and differential scanning calorimetry (DSC) techniques were used. Finally, the fracture surfaces of the tested specimens were examined using a scanning electron microscopy (SEM) analysis. 

## 2. Experimental Details

### 2.1. Materials

The sisal fabric and ramie fibers were provided by Sisalsul (São Paulo, Brazil), the curauá fibers were supplied by the Federal Rural University of Amazonia (UFRA, Amazônia, Brazil) and the glass fibers were provided by Barracuda Advanced Composites (Rio de Janeiro, Brazil). The sisal fabric was used as a base fiber for the composites reinforcement. The approximate thickness and areal density of the sisal fabric were approximately 2.5 mm and 1313 g/m², respectively, according to the specifications of the supplier. The other fibers were stitched in the non-hybrid sisal mats. An epoxy resin, AR260/AH260, supplied by Barracuda Advanced Composites (Rio de Janiero, Brazil), was used as the matrix in this work.

The lignocellulose structure of vegetable fibers consists of three main polymers: cellulose, hemicellulose and lignin. The chemical compositions of the natural fibers vary according to the location, age, species, soil where it was cultivated, rainfall during growth and method of cultivation. The difference in chemical composition between natural fibers can result in different mechanical and thermal properties of the hybrid composites. Table 1 presents the chemical composition and mechanical properties of the natural fibers used in this work [19,21,23,31]. The properties of the glass fiber used were as follow: density (2.55 g/cm³), tensile strength (2000–3500 MPa), Young’s modulus (70–76 GPa) and elongation at break (1.8%–4.8%) [17,21].

### 2.2. Hybrid Fabric Production

**Figure 1:**
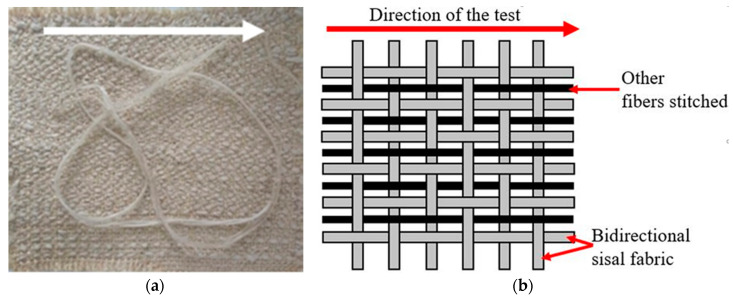
(**a**) Bidirectional sisal fabric and (**b**) schematic drawing of intralaminar configuration.

The fibers used for reinforcement were in yarn form and were stitched in sisal fabric, with a mass fraction of approximately 60%/40% of sisal and other fibers, respectively. The finalized hybrid composite presents longitudinal reinforcement of the other fibers. A schematic drawing of the intralaminar configuration can be seen in Figure 1.

### 2.3. Composite Manufacture

The Sisal+Ramie (S+R), Sisal+Curauá (S+C) and Sisal+Glass (S+G) fiber-reinforced composites were fabricated by the hand lay-up technique using a metallic mold. The cure was performed in a Solab SL-20 hydraulic press (Piracicaba, São Paulo, Brazil), at 80 °C for 6 h. The total weight ratio used in the fabrication of the composites was approximately 30% of fibers and 70% of resin (AR260/AH260 resin was used at a weight proportion of 100 parts of resin for 26 parts of hardener).

### 2.4. Test Methods

#### 2.4.1. Tensile Test

**Figure 2:**
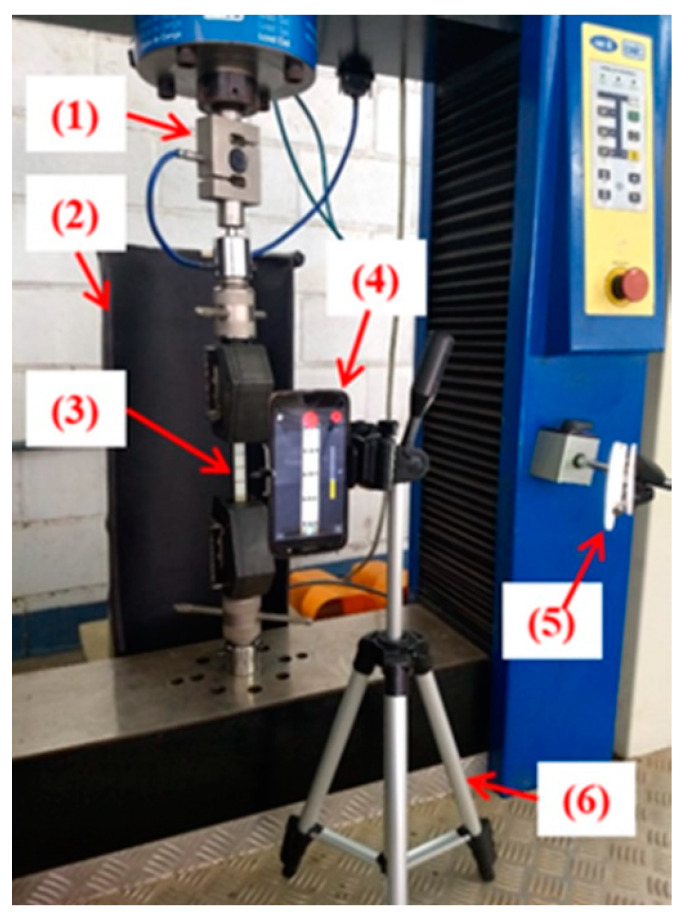
Tensile test set-up. (**1**) Load cell of 20 kN; (**2**) black cardboard to make a contrast in the background of the specimens; (**3**) tensile specimen; (**4**) digital camera of 13 MegaPixel; (**5**) lighting and (**6**) pedestal.

Tensile tests were performed according to ASTM D638 [32], on the EMIC universal testing machine with a load cell of 20 kN, available in the Mechanical Testing Laboratory of the CEFET/RJ (Campus Angra dos Reis, Brazil). The testing speed was 5 mm/min according to the ASTM standard. For the displacement measurements the Digital Image Correlation (DIC) method was used. Five specimens of dimensions 165 mm × 19 mm × 3 mm were tested for each composite type. All the tests were conducted at room temperature (23 °C) and 50% relative humidity. Figure 2 shows the experimental test set-up for the tensile test.

The DIC method is an optical-numerical procedure capable of determining displacement fields through a system that captures images of the specimen surfaces. These images were compared and the elongation was measured during the test. The advantage of this method is that the displacement field is measured without contact with the specimen [33].

#### 2.4.2. Flexural Test

The flexural test was performed according to ASTM D790 [34], using the Instron^®^ 5966 universal testing machine (Norwood, MA, USA) available in the Laboratory of Adhesives and Composites Materials (LADES) of CEFET/RJ Campus Maracanã. The testing speed was 1 mm/min according to ASTM D790-07 standard [34] and a load cell of 1 kN was used. Five specimens of dimensions 127 mm × 12.7 mm × 3 mm were used for each composite type. The span used was 104 mm. The tests were conducted at room temperature (23 °C) and 50% relative humidity. The specimens did not fail and the tests were stopped within 5% of the flexural deformation, according to the ASTM standard.

#### 2.4.3. Impact Test

Impact tests were performed, according to ASTM D6110 [35] and ISO 179 [36], at room temperature (23 °C) and 50% relative humidity, on the INSTRON CEAST 9050 machine available in the Polymer Technology Laboratory (TECPOL) of the Polytechnic Institute of UERJ (IPRJ) (Friburgo, Brazil), with a hammer of 5.4 J. Five specimens of dimensions 110 mm × 12.7 mm × 3 mm were tested for each composite type.

#### 2.4.4. Scanning Electron Microscopy (SEM) Analysis

The cross-sections of specimens were observed using a high-resolution scanning electron microscopy (SEM), FEI Inspect™ with a 20 KV voltage acceleration available in the Nanotechnology Characterization Laboratory of the National Institute of Technology (CENANO/INT, Rio de Janeiro, Brazil). The samples were coated with a gold layer.

#### 2.4.5. Thermogravimetric Analysis (TGA)

The TGA was carried out in a NETZSCH TG 209F3 Tarsus equipment available in the LADES (CEFET/RJ, Rio de Janeiro, Brazil). Each sample was tested in the temperature range of 30–600 °C at a constant rate of 10 °C min^−1^ under nitrogen (N_2_) atmosphere. About 20 mg of each composite sample were used to make the measurements. ASTM E1131 standard was used [37].

#### 2.4.6. Differential Scanning Calorimetry (DSC)

The temperature dependent transformations of the composite samples were carried out in a NETZSCH DSC 200F3 Maia equipment, available in the LADES (CEFET/RJ, Rio de Janeiro, Brazil). The experiment was conducted with a heating rate of 20 K min^−1^, in the temperature range of 30–500 °C with a nitrogen (N_2_) flux of 50 mL min^−1^. About 20 mg of each sample were used. ASTM D3418 standard was used [38].

## 3. Experimental Results and Discussion

### 3.1. Mechanical Characterization

#### 3.1.1. Tensile Test

**Figure 3:**
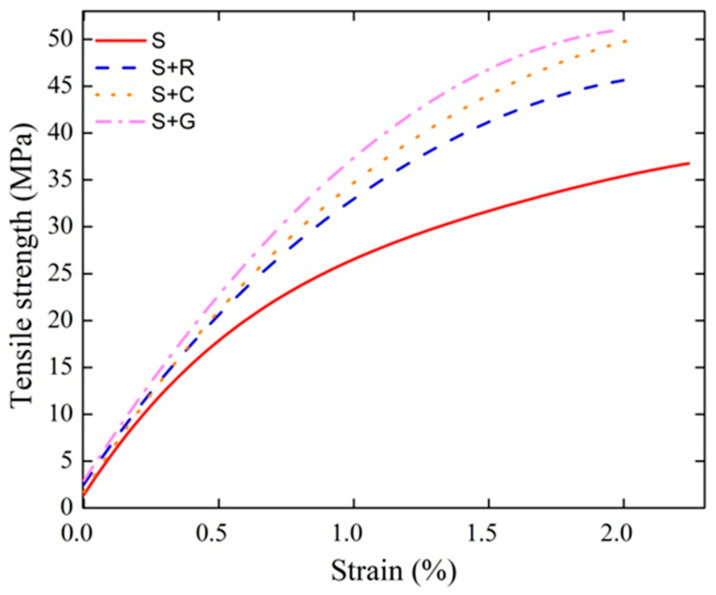
Representative tensile stress–strain curves as a function of hybridization.

Representative true tensile stress–strain curves are shown in Figure 3. From these curves the tensile data was calculated (i.e., tensile strength, the Young’s modulus and tensile failure strain).

Table 2 presents the tensile data obtained from the tensile tests of the hybrid composites studied. It can be seen that the hybridization process improved the tensile properties of all types of hybrid composites studied when compared to the non-hybrid sisal composite (S). For instance, the tensile strength of S+R composites increased by 24.83% compared to S composite, while the tensile strength of S+C and S+G composites increased by 34.98% and 43.99%, respectively. It should be noted that S+C have relatively similar tensile properties as the S+G composite (50.01 ± 2.62 vs. 53.35 ± 4.04 MPa). The results are in agreement with the literature [17], where the hybridization of non-hybrid jute composites also provided an improvement of the tensile strength. It is well known that, in hybrid composites, the properties are primarily dependent on the elongation at break and the modulus of elasticity of the single reinforcing fibers. The mechanical properties of fibers (i.e., modulus and ultimate tensile strength) are related not only to the chemical composition of the fiber but also to its internal structure. The lower tensile properties of ramie compared to the curauá fibers as found in a previous study [21] explain the lower improvement in tensile strength of the S+R composite compared to S+C. On the other hand, the modulus of glass fiber is comparatively higher than that of the vegetable fibers used here, whereas the extensibility of glass is low compared to the sisal and curauá fibers [17].

Similar to the tensile strength, the Young’s modulus of the composites studied here was also affected by the hybridization. The Young’s modulus increased by approximately 14.01% for S+R, by approximately 32.14% for S+C and 50.00% for S+G composites when compared to the non-hybrid sisal composites. The higher increase in Young’s modulus of S+G composite was due to the higher modulus of glass fiber compared to the natural fibers.

The failure strain was also affected by the hybridization. It is known that in a hybrid composite, failure is directly related to the strain at break of the individual reinforcing fibers [39] and it was shown in the literature that the strain at break of the glass fiber (2.5%) is lower compared to the curauá fiber (4.5%) [40], which may explain the lower strain failure of S+G composites compared to S+C. These results are in agreement with the literature [19,23,31].

#### 3.1.2. Flexural Test

Table 3 presents the data obtained from the flexural tests as a function of hybridization. In accordance to the ASTM D790 the tests were stopped within 5% of the flexural deformation, when the specimens did not fail. It can be seen that the flexural strength and modulus of the composites increase with the incorporation of fibers in the non-hybrid sisal composite. It is known that, in flexural testing, various mechanisms such as tension, compression and shearing take place simultaneously. In general, the flexural properties of composites are mainly determined by the compression properties of the upper layer and the tensile properties of the lower layer of the composite. For the cases studied here, the flexural strength increased due to the increased resistance to shearing of the hybrid composites compared to non-hybrid sisal composite [39,41].

The flexural strength of S+R composites increased by approximately 37.81% compared to non-hybrid sisal composites, while S+C and S+G presented an increase by approximately 51.63% and 58.54%, respectively. As expected, the S+G composite presented the best flexural strength (82.6 ± 4.77 MPa), which is explained by the better adhesion of synthetic fiber to epoxy matrices [41]. Cavalcanti et al. [17] also found that the jute + glass fiber composite presented the best flexural strength (166.92 ± 35.33 MPa), compared to jute + sisal and jute +curauá composites. 

The flexural modulus was also affected by the hybridization. The flexural modulus of S+R composites increased by approximately 40.64% compared to non-hybrid sisal composites, while S+C and S+G presented an increase by approximately 57.91% and 63.31%, respectively. 

#### 3.1.3. Impact Test

Table 4 presents the data obtained from the impact tests. It is well known that the impact strength of the composite depends on the properties of the individual fibers used for the preparation of hybrid intralaminar composite laminates and on the interfacial adhesion between fiber and matrix. For the S+C composites an increase of impact resistance of approximately 47.28% was found, while for S+G composites an increase of approximately 50.90% was observed, when compared to non-hybrid sisal composites. Ramesh et al. [42] also reported an increased impact strength due to the hybridization with glass and jute fiber into the sisal composites. Cavalcanti et al. [17] studied the effect of hybridization on the impact properties of intralaminar hybrid epoxy composites based on the jute and found that the energy absorption of jute + sisal and jute + curauá hybrid composites presented values close to those of the jute + glass fiber composites.

On the other hand, S+R composites presented an insignificant increase of impact resistance (i.e., 2.59%) when compared to the non-hybrid sisal composites. This may be explained by the fact that the impact strength of the composite depends on the properties of the individual fibers used for preparation of hybrid intralaminar composites [43]. Lima et al. [21] found that the tensile strength of ramie was 212.38 MPa, while for the sisal it was 398.48 MPa. They also showed that the diameter of ramie fiber (95 ± 5 µm) was smaller compared to sisal fiber (150 ± 1 µm), which may explain the lower increase in impact strength compared to other fibers used.

#### 3.1.4. SEM Analysis

SEM images of the tensile and impact fractured surfaces were taken to analyze the fiber–matrix interaction and the fracture behavior.

**Figure 4:**
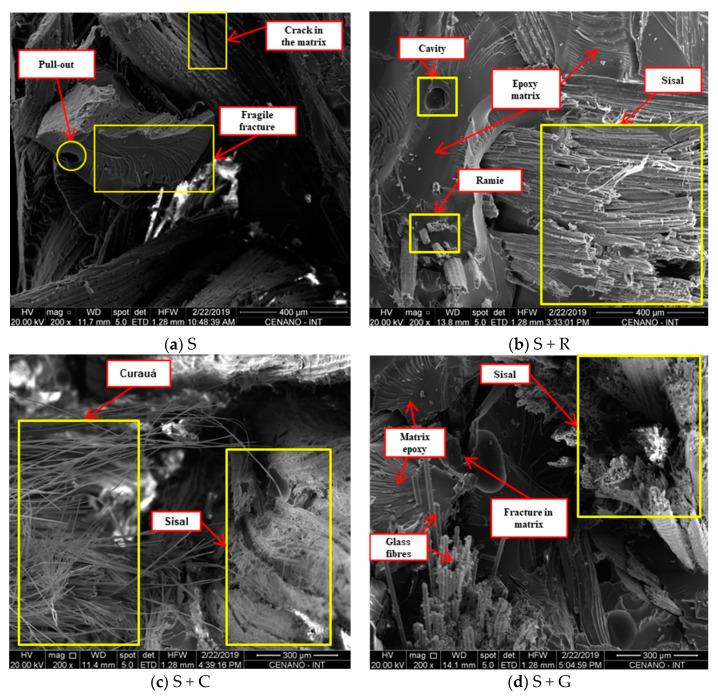
SEM images of fracture surface of tensile specimens as a function of hybridization. (**a**) S; (**b**) S+R; (**c**) S+C and (**d**) S+G.

Figure 4 shows the fracture surface of the tensile specimens as a function of hybridization. A brittle fracture of the matrix and fiber breakage near the matrix could be observed. It is known that the fracture of fibers is common in composites with strong interfacial bond. It can be seen that some fibers were released by the ‘‘pull-out’’ mechanism, which might indicate the lack of fiber/matrix adhesion (Figure 4a) and other fibers were broken close to the matrix (Figure 4b,d). The presence of some voids can be also observed.

**Figure 5:**
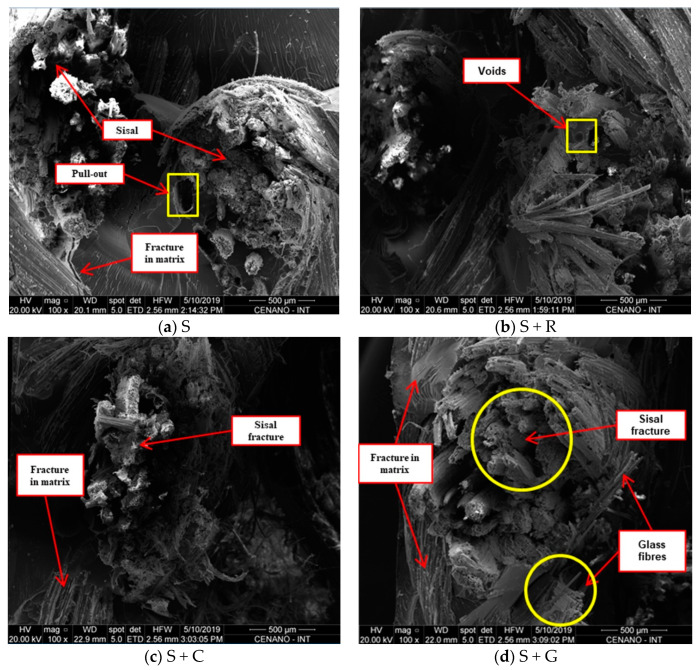
SEM images of fracture surface of composites as a function of hybridization after the impact tests (**a**) S; (**b**) S+R; (**c**) S+C and (**d**) S+G.

Figure 5 presents the fracture surface of impact test specimens. It is known that the impact energy is dissipated by debonding, fiber and/or matrix fracture and fiber pull out. Fiber fracture dissipates less energy compared to fiber pull out. The SEM images show evidence of fiber fracture and few voids can be seen in the matrix due to fiber pull-out. Fiber pull-out is a sign of a weak bond, while the fracture of fibers is common in composites with strong interfacial bond.

### 3.2. Thermal Analysis

#### 3.2.1. Thermogravimetric Analysis (TGA) 

**Figure 6:**
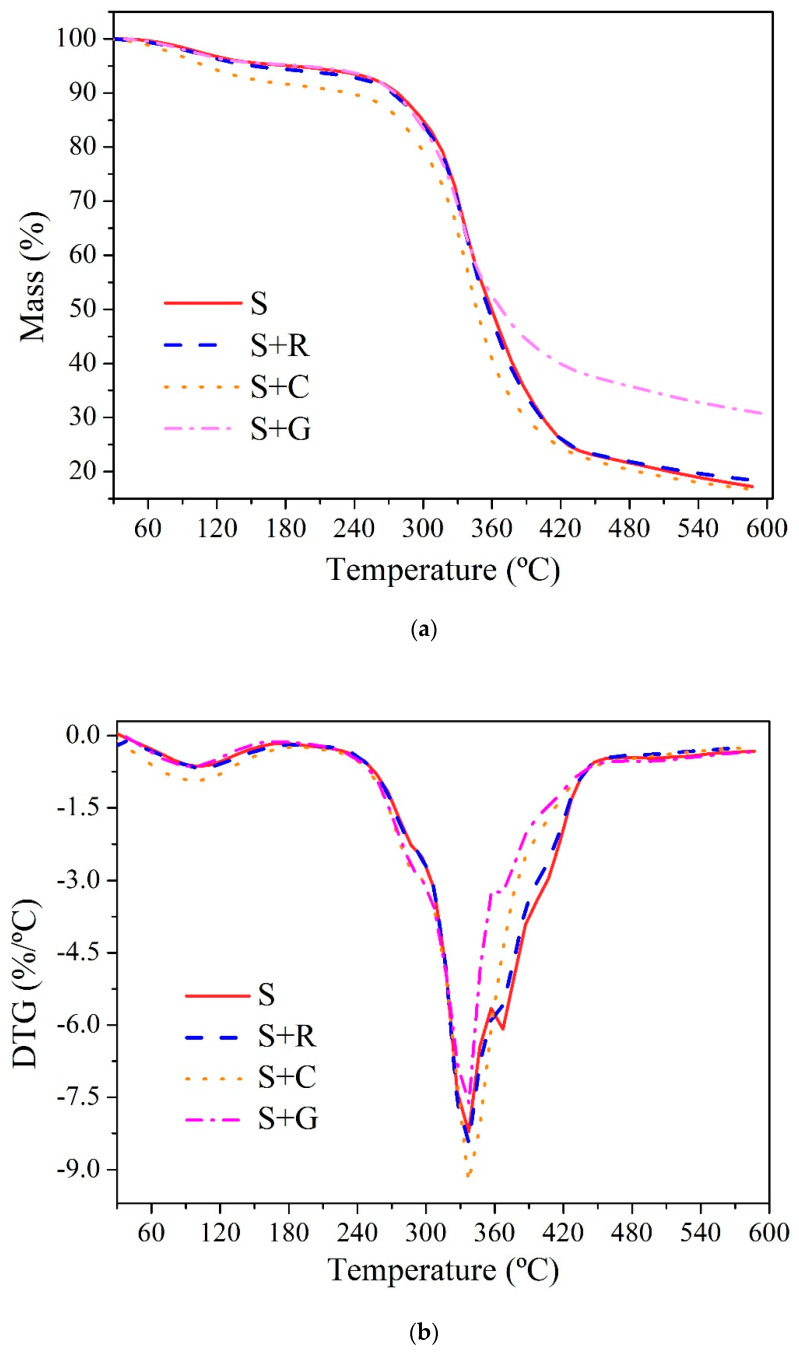
Thermal analysis typical curves for the hybrid composites: (**a**) TG and (**b**) DTG.

Thermogravimetric analysis (TGA) measurements were performed to obtain information on the thermal stability of the composites. Figure 6 shows the composite thermal degradation as a function of hybridization. It is well known that when the material is degraded, it loses mass in the form of volatiles [31]. From Figure 6a it can be seen that the TG curves showed similar tendencies. It should be noted that the S+G composites show low mass loss at higher temperatures when compared to natural hybrid composites. This was expected, since the decomposition temperature of glass fibers is higher compared to the decomposition temperature of the natural fibers.

From a practical point of view, the thermal stability of the natural fiber composites is related to the onset of a massive weight loss. This is clearly observed as a sharp downward inclination in the TG curve, which can be observed at 240–420 °C in Figure 6a.

Figure 6b shows the DTG curves for sisal based hybrid composites, where two main stages of decomposition could be observed: a first stage with a slight mass loss in the range of 30–150 °C due to the release of humidity retained in the fibers and a second stage, which occurred between 260 and 420 °C due to the pyrolysis process. This second stage is also, where the main lignocellulosic constituents of the fibers thermal degradation occurs (270–350 °C) [44]. The major degradation peaks appeared at 337 °C and they were almost the same for pure sisal and the hybrid composites investigated here. S+G composites revealed lower speed of mass loss compared to the other samples. Gañán et al. [44] showed TG/DTG curves for epoxy (DGEBA/TETA) composites reinforced with 30 wt % of sisal fibers with different surface treatments of the fibers (i.e., untreated or mercerized, silanized or silanized with previous mercerization). They showed that the composites present a region at 210–350 °C associated with the sisal fiber constituents’ decomposition. 

#### 3.2.2. DSC

The DSC measures the temperature and heat flux associated with material transitions as a function of temperature and time. Qualitative and quantitative information on physical and chemical changes involving endothermic (heat absorption) and exothermic (heat released) processes is provided by this technique. The exothermic and endothermic peaks, and magnitudes indicate the thermal phase transformation of the composites [45,46].

**Figure 7:**
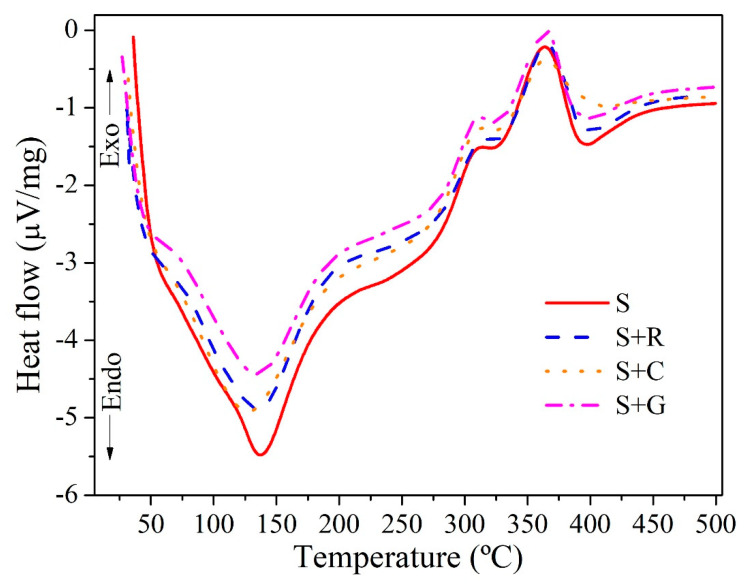
Differential scanning calorimetry (DSC) curves of composites as a function of hybridization.

Figure 7 presents the DSC curves for the hybrid composites studied, where two events predominated (endothermic and exothermic). The endothermic peak appeared from room temperature to 140 °C and is related to the dehydration process of the composite samples [30,45]. The S+R and S+C composites presented a slightly lower endothermic peak value compared to non-hybrid S composites (approximately 136.5 and 137.8 compared to 140.5 °C). This can be explained by the fact that composites with higher natural fiber content shows a higher water absorption [5]. Furthermore, the exothermic peak found around 365 °C is attributed to the decomposition of cellulose/lignin and the matrix [47,48,49]. 

The DSC data for each composite as a function of hybridization is shown in Table 5. 

## 4. Conclusions

In this research, the effects of hybridization on the mechanical and thermal properties of natural intralaminar hybrid composites based on sisal were investigated. The following conclusions can be drawn:

- The hybridization process improved the tensile properties of the non-hybrid sisal fiber based composites. For S+C and S+G composites, the increase in tensile strength was by 34.98% and 43.99%, respectively, compared to non-hybrid sisal composites, while for S+R composite the improvement in strength was by approximately 24.83%. The Young’s modulus increased for S+R (by approximately 14.01%), S+C (by approximately 32.14%) and S+G (by approximately 50.00%) composites when compared to the non-hybrid sisal composites.
- The flexural strength and modulus of the hybrid composites were also affected by the incorporation of fibers. S+C and S+G composites had a flexural strength increase of approximately 51.63% and 58.54%, respectively, compared to non-hybrid sisal composites, while for S+R composite the increase in flexural strength was by approximately 37.81%. The best flexural modulus was found for the S+G composite (an increase by approximately 63.31% compared to non-hybrid sisal composites).
- The hybridization improved the impact properties for S+C (an increase of approximately 47.28%) and S+G composites (an increase of approximately 50.90%), while the impact resistance of S+R had an insignificant increase (approximately 2.59%) when compared to non-hybrid sisal composites.
- The SEM analysis showed a brittle fracture of the epoxy matrix and fibers breakage near the matrix.
- The DTG curves showed two main stages of decomposition: a first stage with a slight mass loss in the range of 30–150 °C due to the removal of the moisture from the composites and a second stage, which occurs between 240 and 420 °C due to the pyrolysis process. The major degradation peak appears from the DTG plots to occur at around 337 °C for all composites. This shows that the hybridization did not affected the thermal stability of the composites.
- The DSC analysis showed that the S+R and S+C composites presented a slightly lower endothermic peak value compared to non-hybrid S.

Natural fibers can be combined to produce hybrid composites, which take full advantage of the best properties of each component. Hybrid natural fiber-reinforced composites have a potential to replace glass fiber-reinforced composites in applications that do not require very high load bearing capabilities.

## Figures and Tables

**Table 1:** Chemical composition, physical and mechanical properties of the natural fibers studied [19,21,23,31].

Properties	Sisal	Ramie	Curauá
Density (g/cm³)	1.30	1.20	1.33
Diameter (µm)	150 ± 1	95 ± 5	46 ± 13
Tensile strength (MPa)	398.48 ± 83.70	212.32 ± 7.28	1929.8 ± 249.5
Young’s modulus (GPa)	61.99 ± 25.30	30.39 ± 10.80	87.23 ± 15.40
Elongation at break (%)	2.74 ± 0.70	2.62 ± 0.20	3.94 ± 0.60
Cellulose (wt %)	67–78	68.6–91	70.7–73.60
Hemicellulose (wt %)	10–14	5–16.60	9.9
Lignin (wt %)	8–11	0.6–0.70	7.5–11.10

**Table 2:** Tensile tests data.

Composites	Tensile Strength (MPa)	Young’s Modulus (GPa)	Strain (%)
S	37.05 ± 2.78	4.20 ± 0.61	2.17 ± 0.27
S+R	46.25 ± 2.27	4.79 ± 0.60	2.10 ± 0.29
S+C	50.01 ± 2.62	5.55 ± 1.01	2.39 ± 0.14
S+G	53.35 ± 4.04	6.30 ± 1.41	2.20 ± 0.36

**Table 3:** Flexural test data.

Composites	Flexural Strength (MPa)	Flexural Modulus (GPa)
S	52.1 ± 6.44	2.78 ± 0.49
S+R	71.8 ± 9.80	3.91 ± 0.43
S+C	79.0 ± 0.74	4.39 ± 0.71
S+G	82.6 ± 4.77	4.54 ± 0.23

**Table 4:** Impact tests data.

Composites	Impact Resistance (J/m)
S	506.1 ± 69.40
S+R	519.2 ± 50.26
S+C	745.4 ± 37.54
S+G	763.7 ± 46.81

**Table 5:** DSC data of the composites studied.

Composite	Endothermic Peak (°C)	Exothermic Peak (°C)
S	140.5	362.9
S+R	136.5	364.2
S+C	137.8	367.6
S+G	144.2	365.5
